# Supplementary figures and images for: Artificial substrata increase pond farming density of grass carp (Ctenopharyngodon idella) by increasing the bacteria that participate in nitrogen and phosphorus cycles in pond water
Source: PeerJ. 2019 Oct 14;7:e7906. doi: 10.7717/peerj.7906 (PMC6796960; doi:10.7717/peerj.7906)

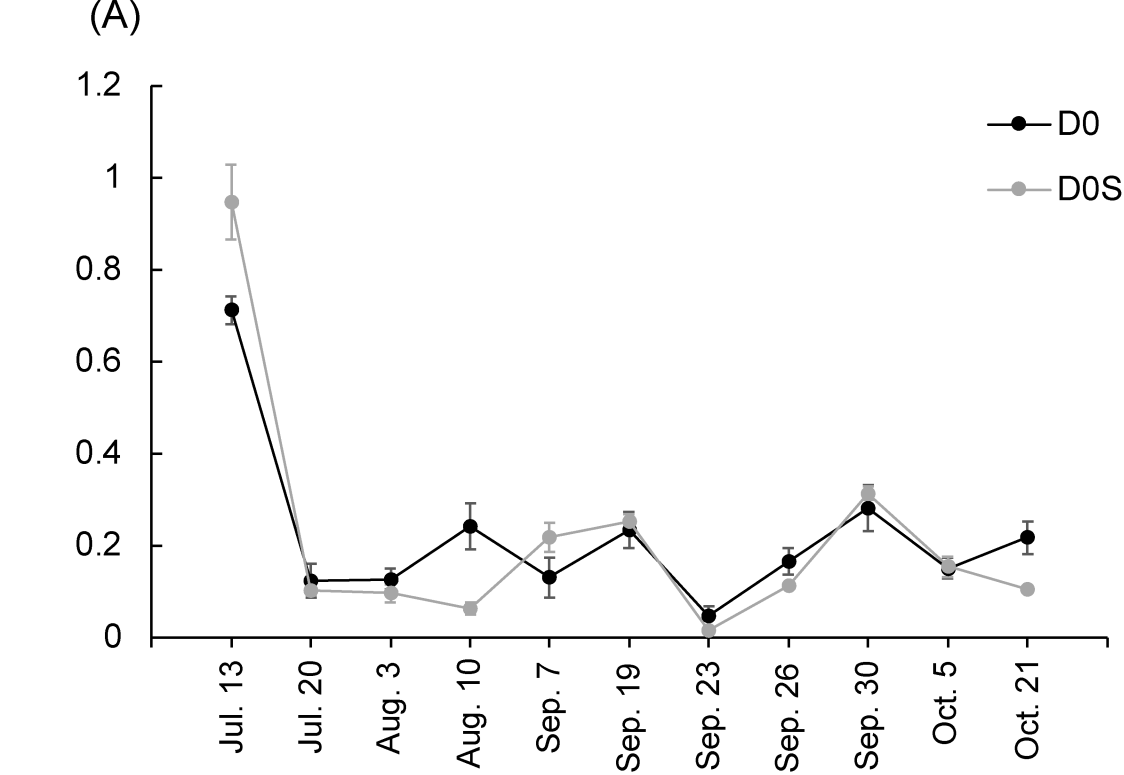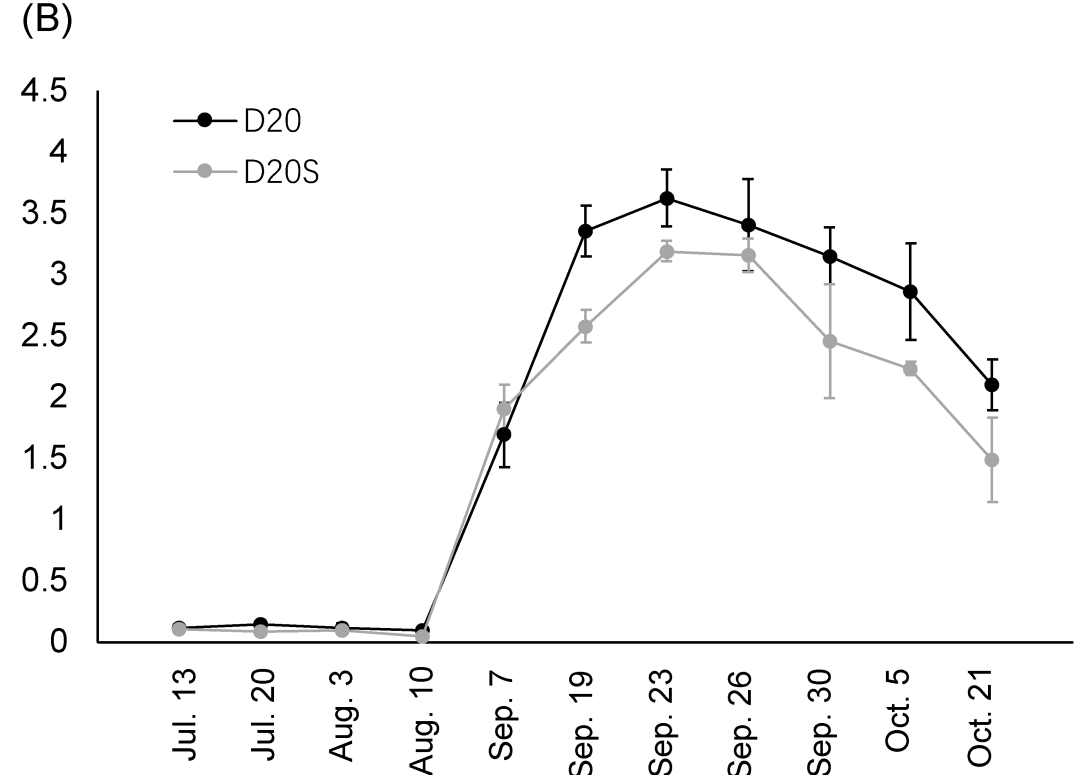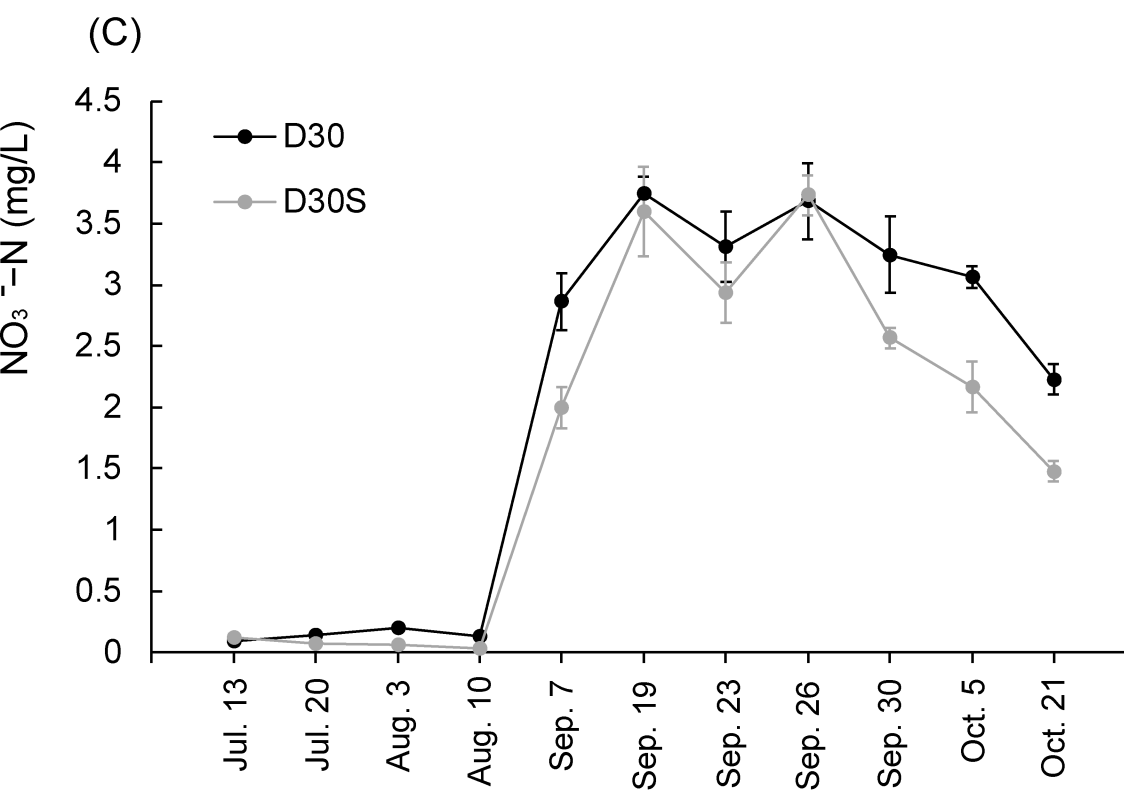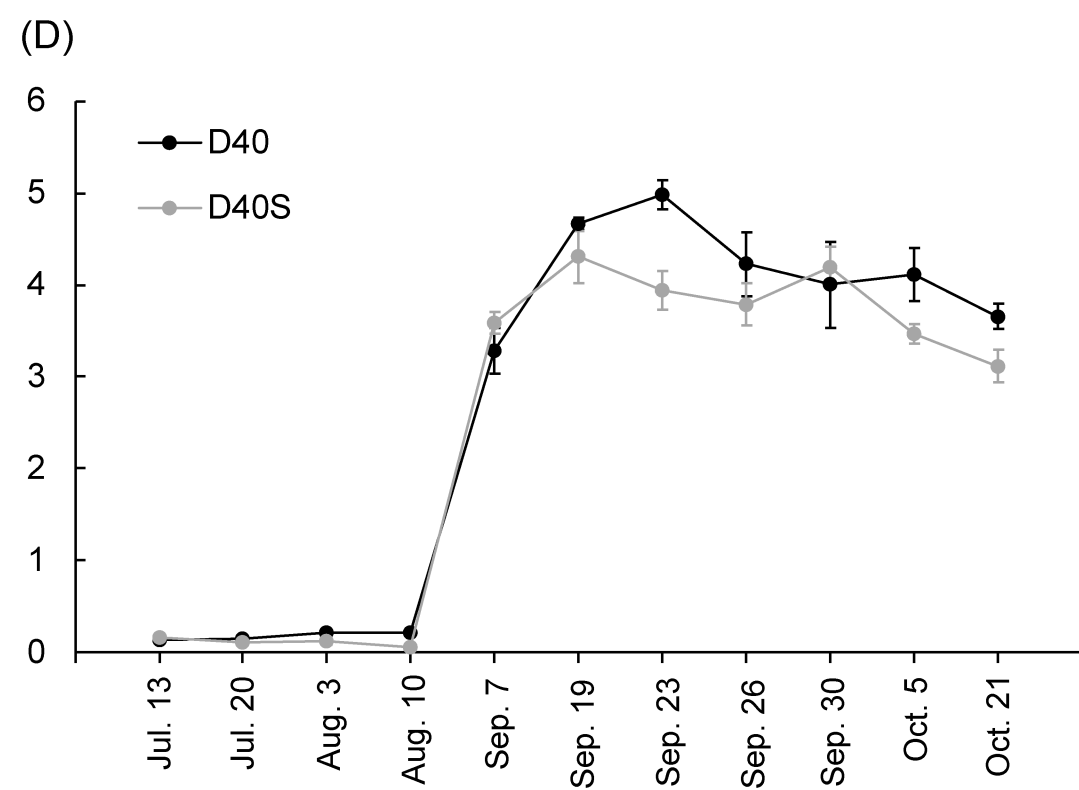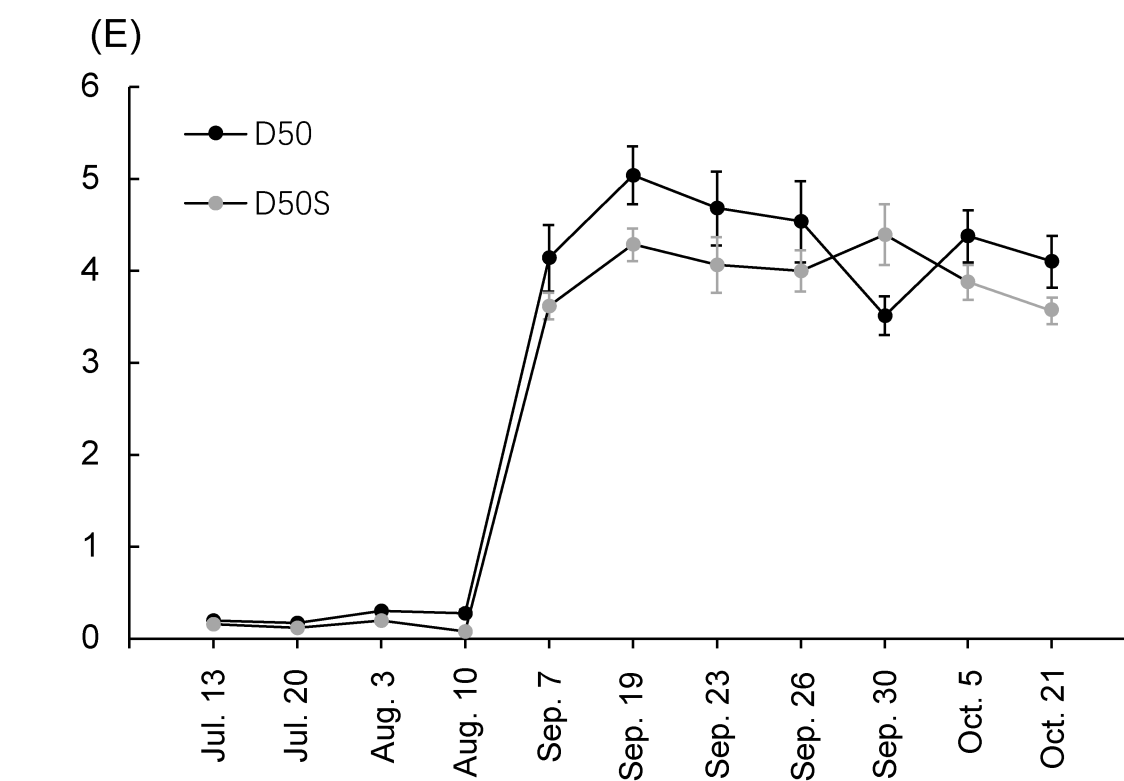

Sampling date

Supplement: Figure S1 — D0, D20, D30, D40, and D50 indicate the pond did not contain the artificial substratum, including 0, 20, 30, 40, and 50 individuals, respectively. D0S, D20S, D30S, D40S, and D50S indicate the pond contained the artificial substratum, including 0, 20, 30, 40, and 50 individuals, respectively. [file peerj-07-7906-s002.pdf]

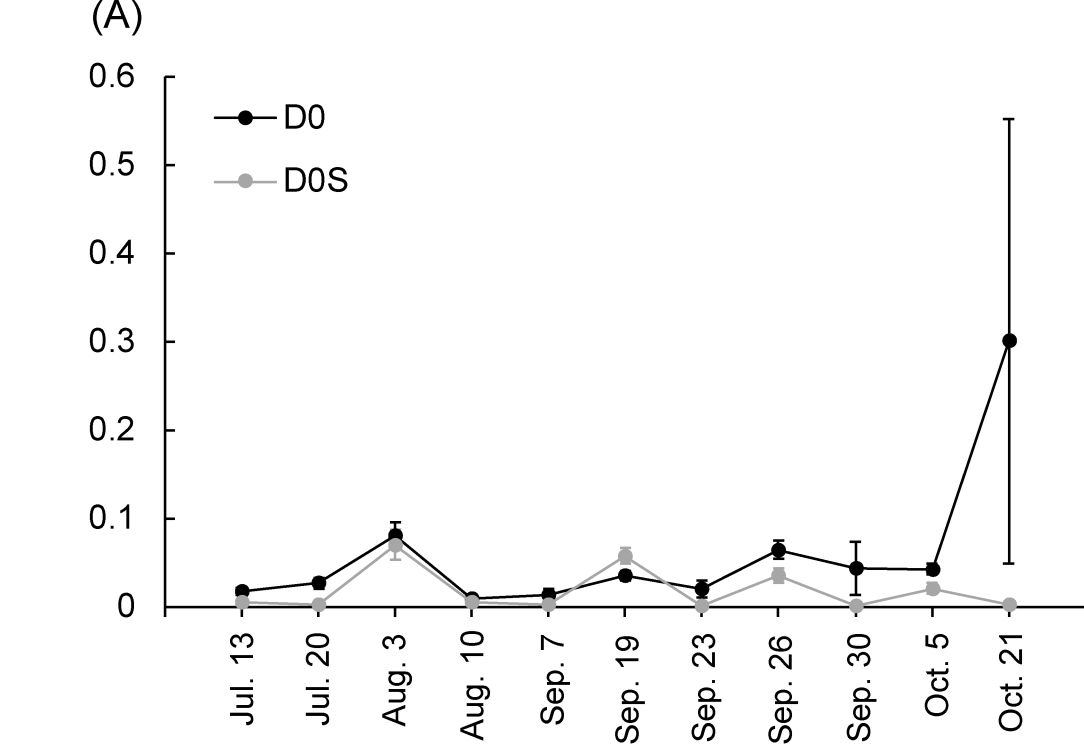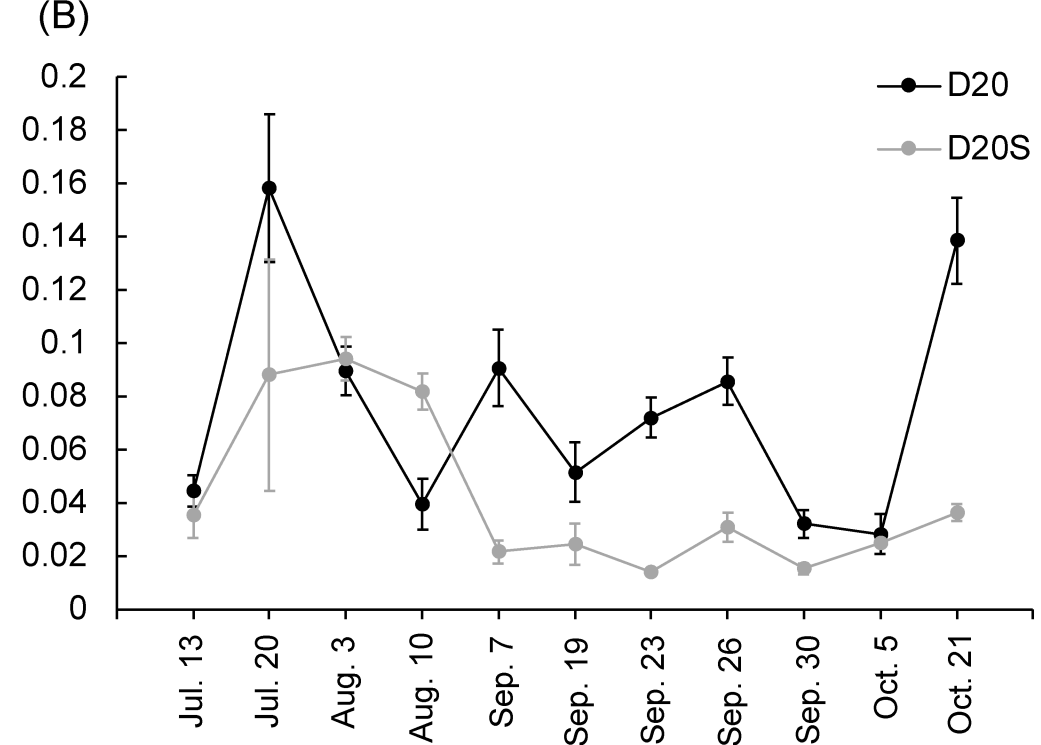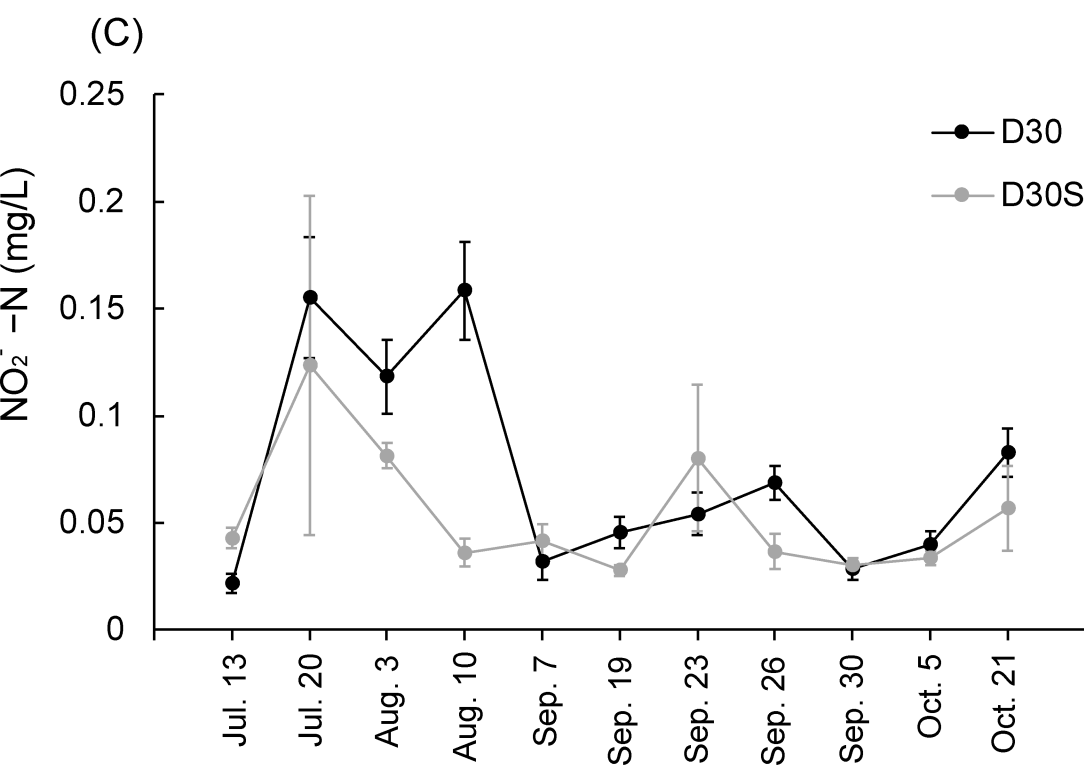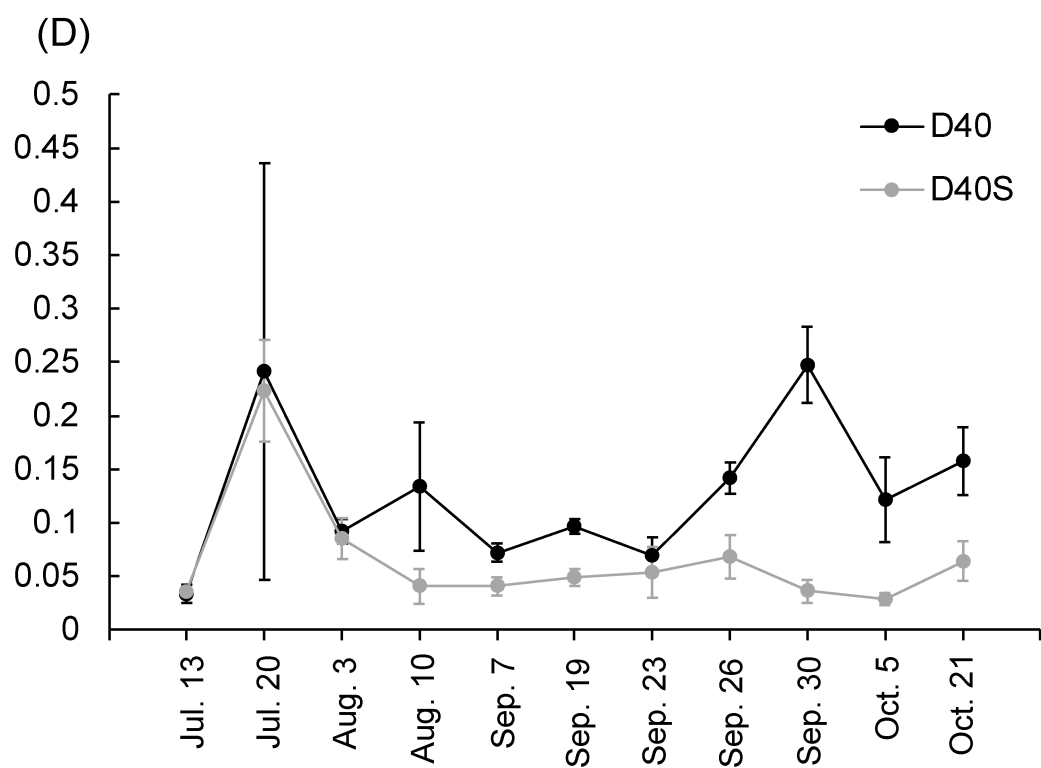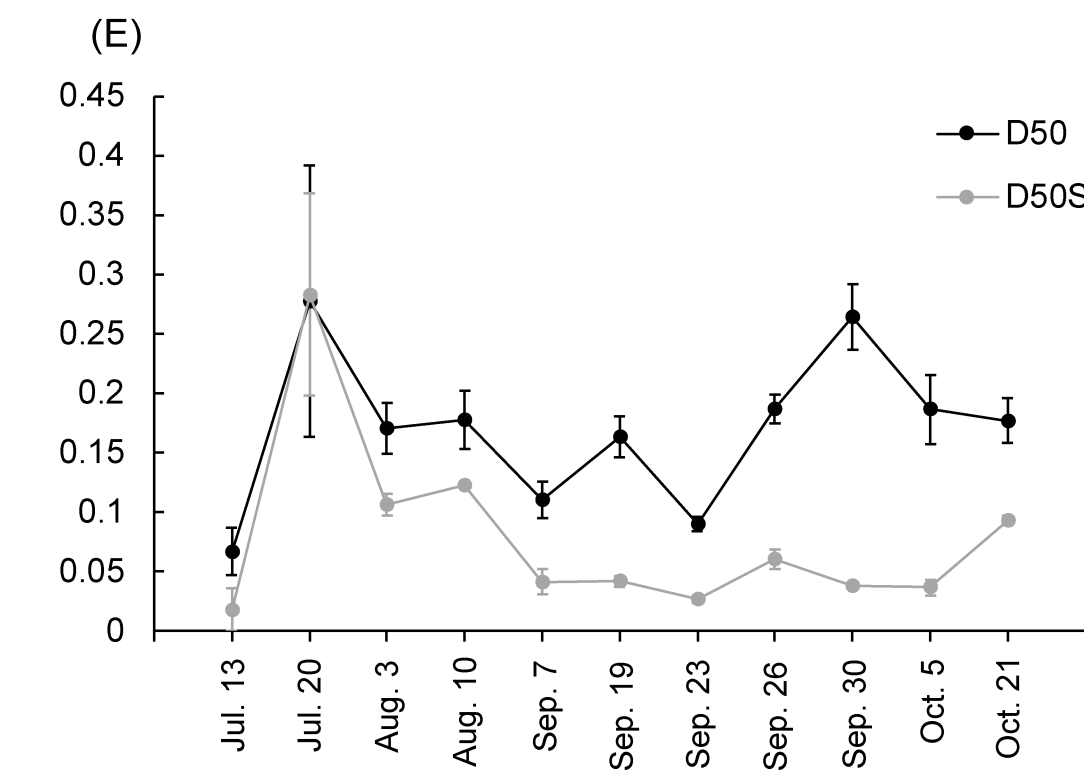

Sampling date

Supplement: Figure S2 — D0, D20, D30, D40, and D50 indicate the pond did not contain the artificial substratum, including 0, 20, 30, 40, and 50 individuals, respectively. D0S, D20S, D30S, D40S, and D50S indicate the pond contained the artificial substratum, including 0, 20, 30, 40, and 50 individuals, respectively. [file peerj-07-7906-s003.pdf]

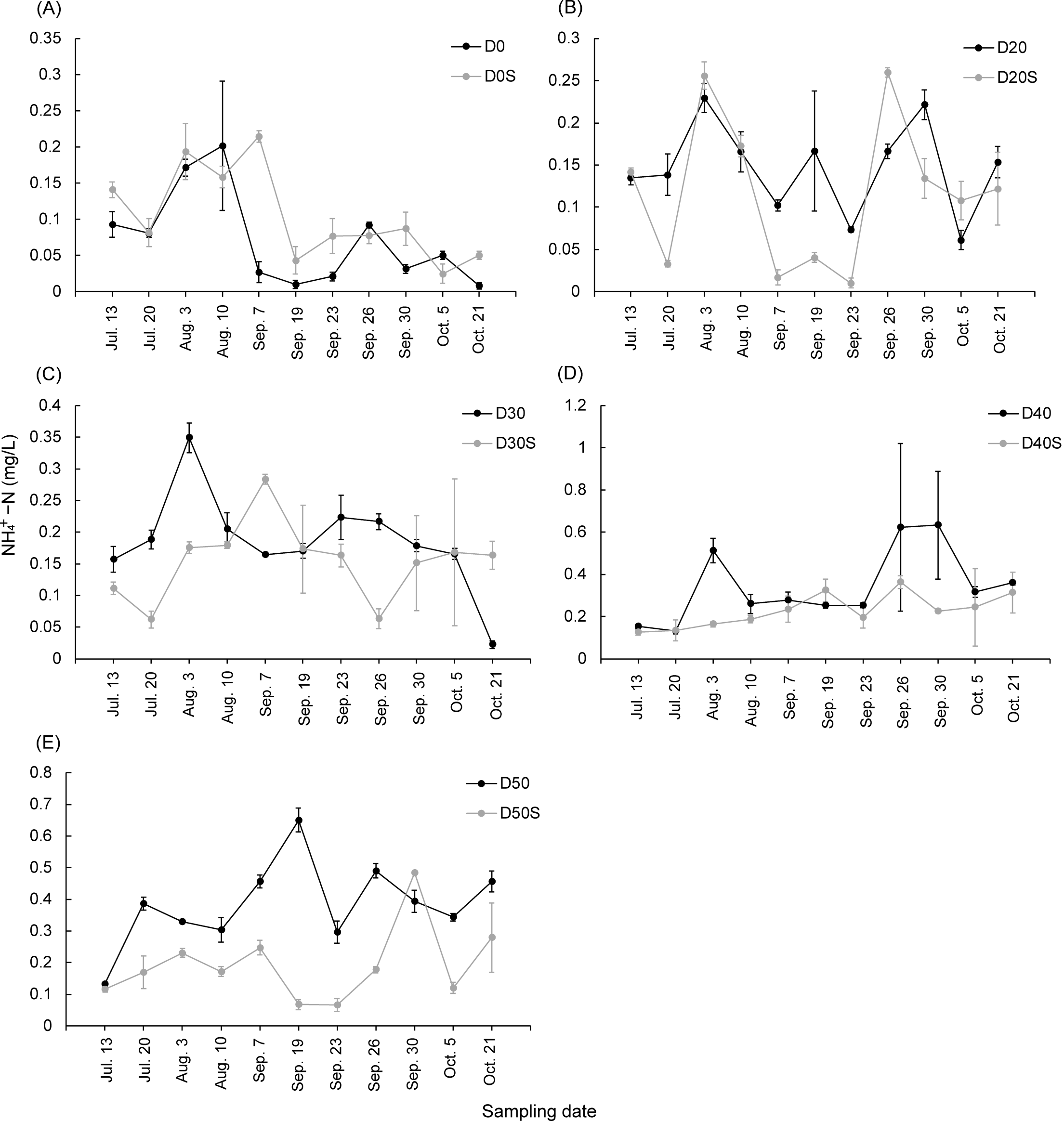

Supplement: Figure S3 — D0, D20, D30, D40, and D50 indicate the pond did not contain the artificial substratum, including 0, 20, 30, 40, and 50 individuals, respectively. D0S, D20S, D30S, D40S, and D50S indicate the pond contained the artificial substratum, including 0, 20, 30, 40, and 50 individuals, respectively. [file peerj-07-7906-s004.pdf]

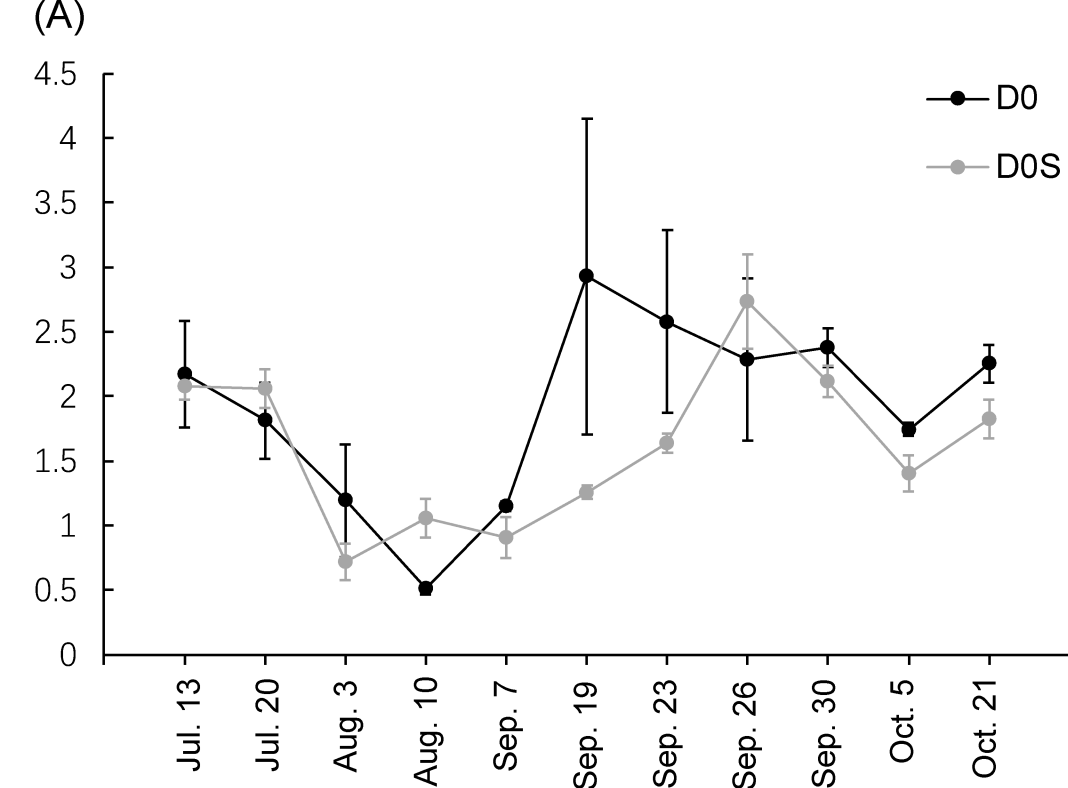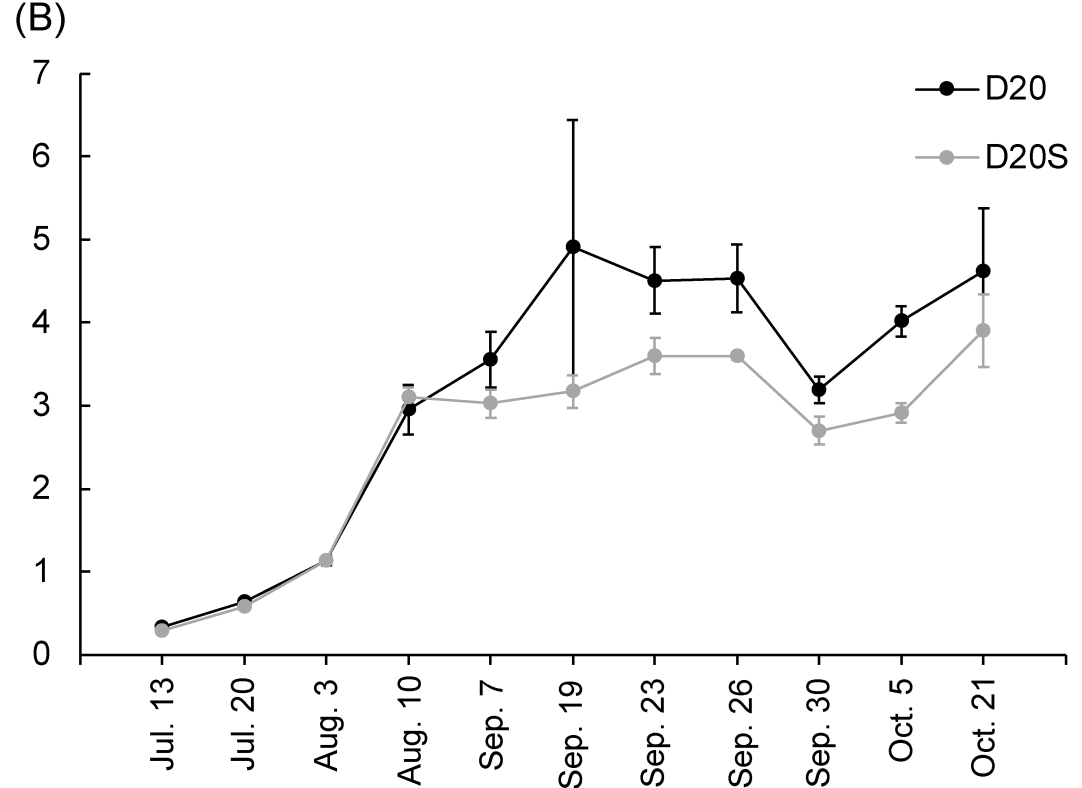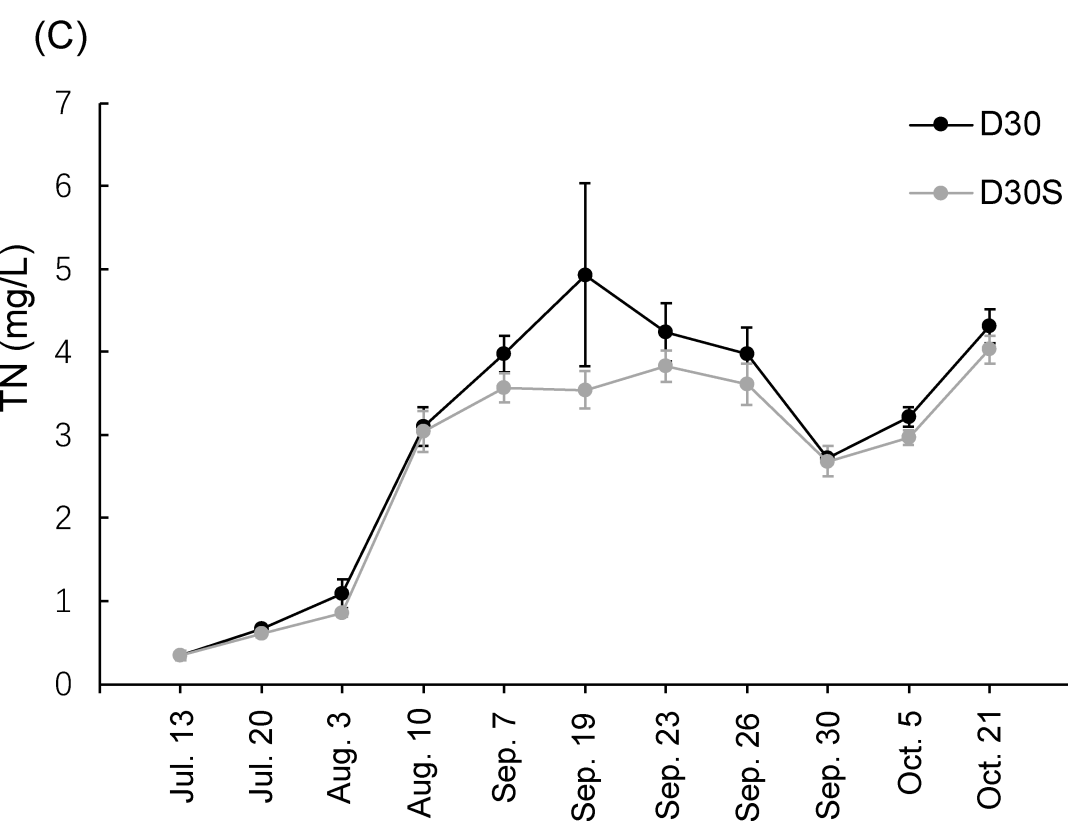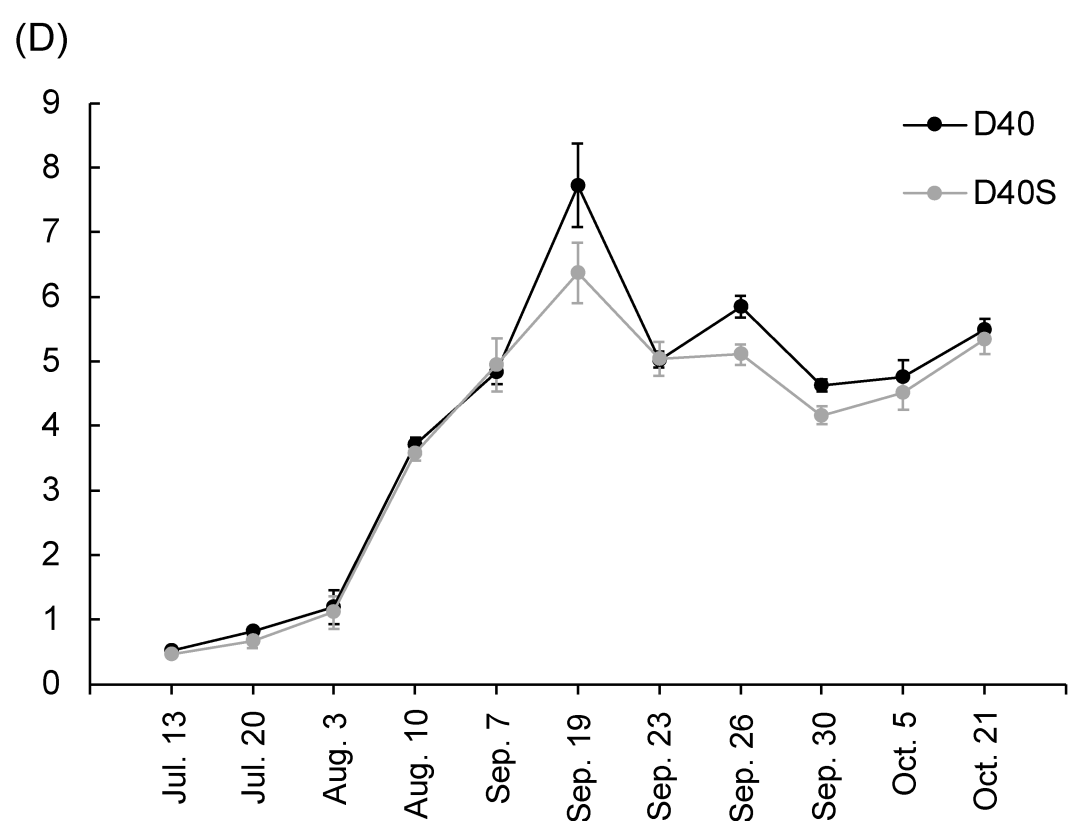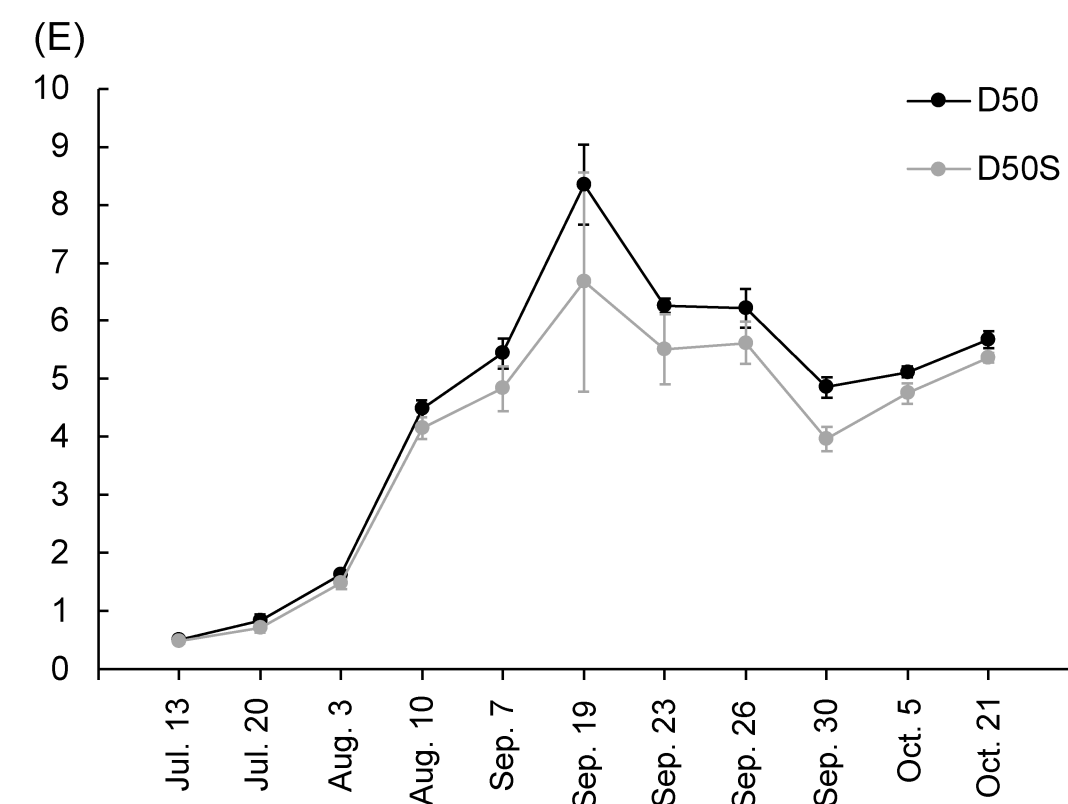

Sampling date

Supplement: Figure S4 — D0, D20, D30, D40, and D50 indicate the pond did not contain the artificial substratum, including 0, 20, 30, 40, and 50 individuals, respectively. D0S, D20S, D30S, D40S, and D50S indicate the pond contained the artificial substratum, including 0, 20, 30, 40, and 50 individuals, respectively. [file peerj-07-7906-s005.pdf]

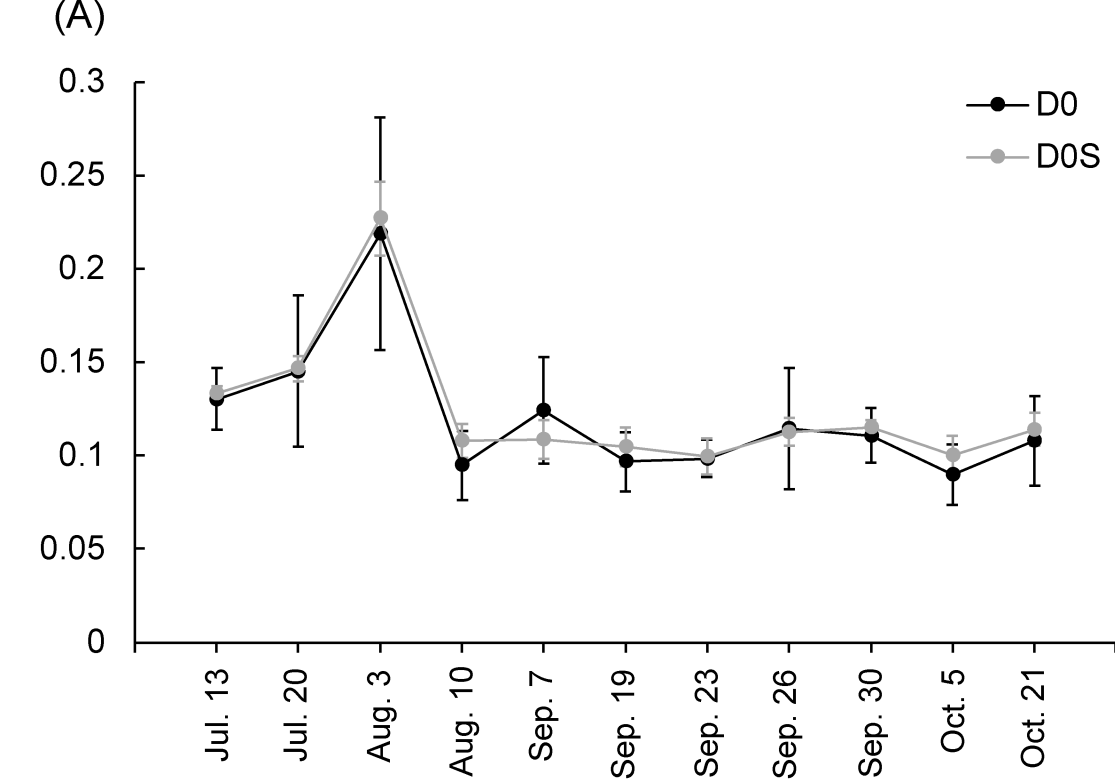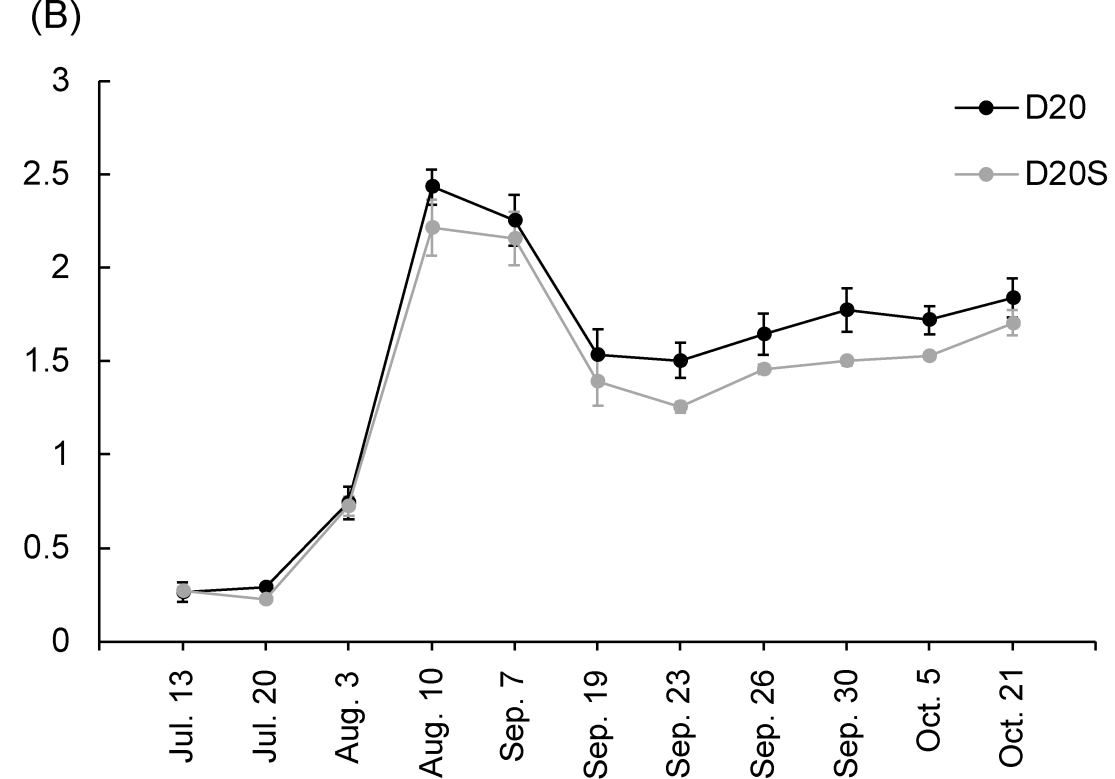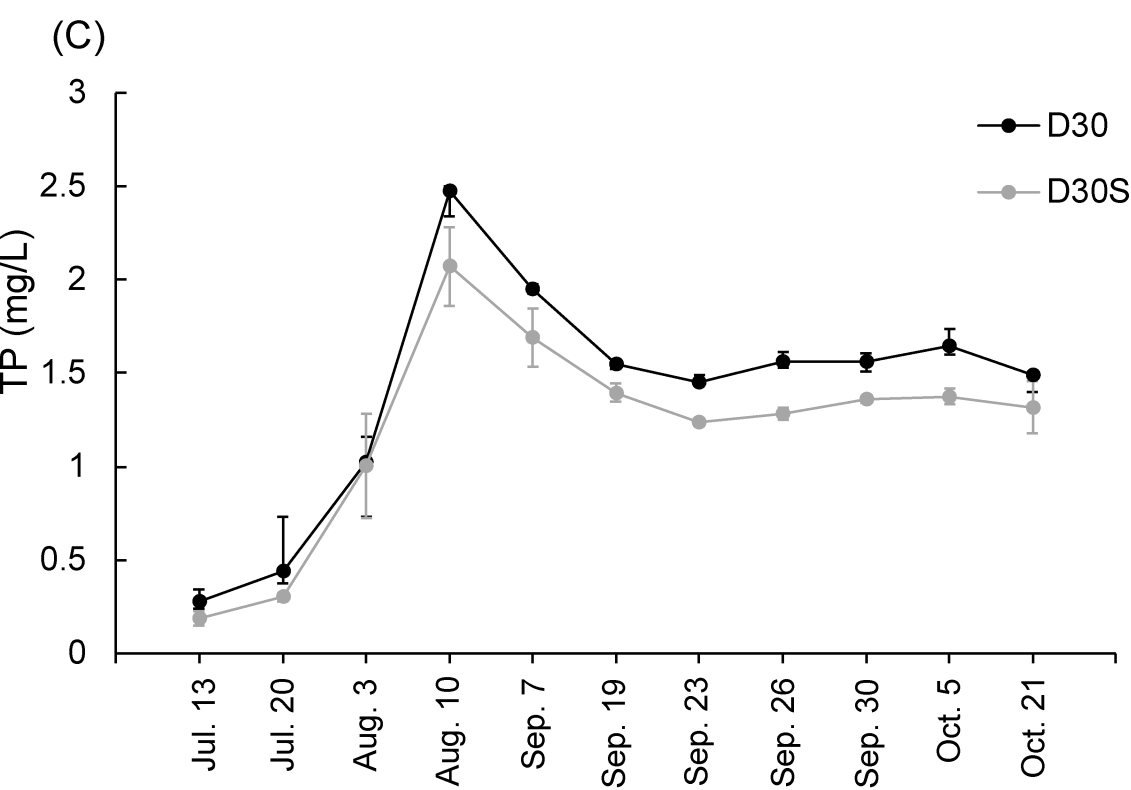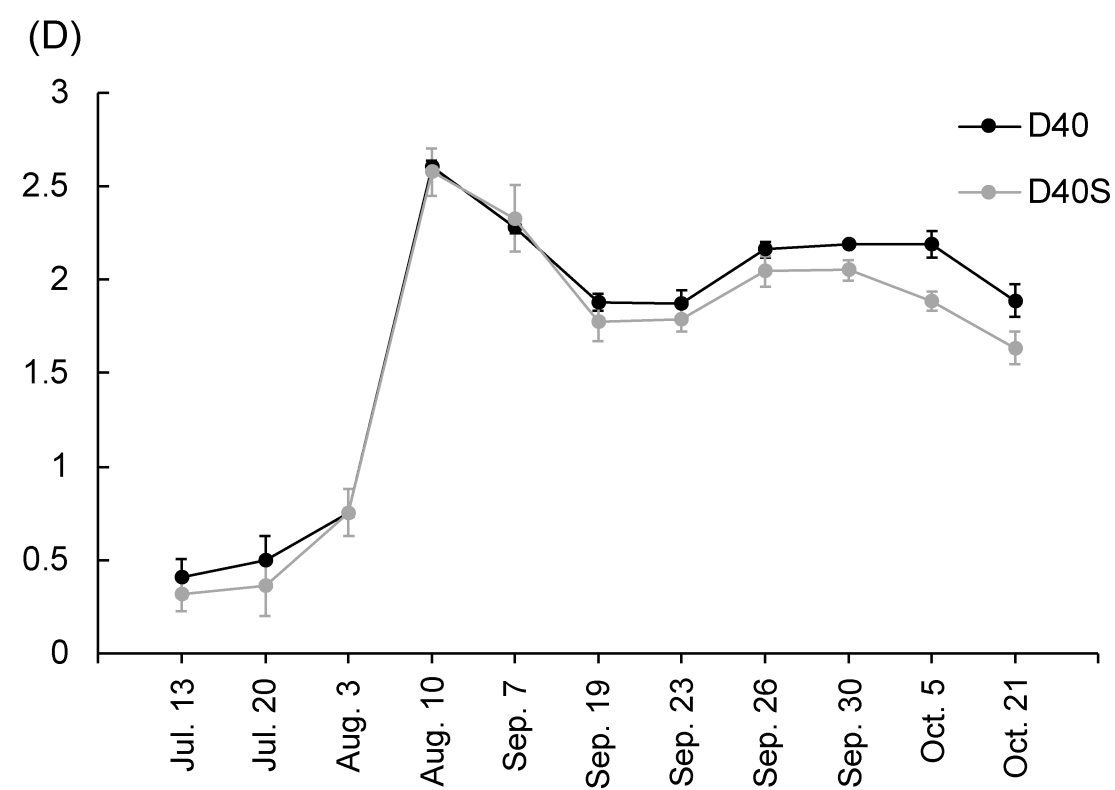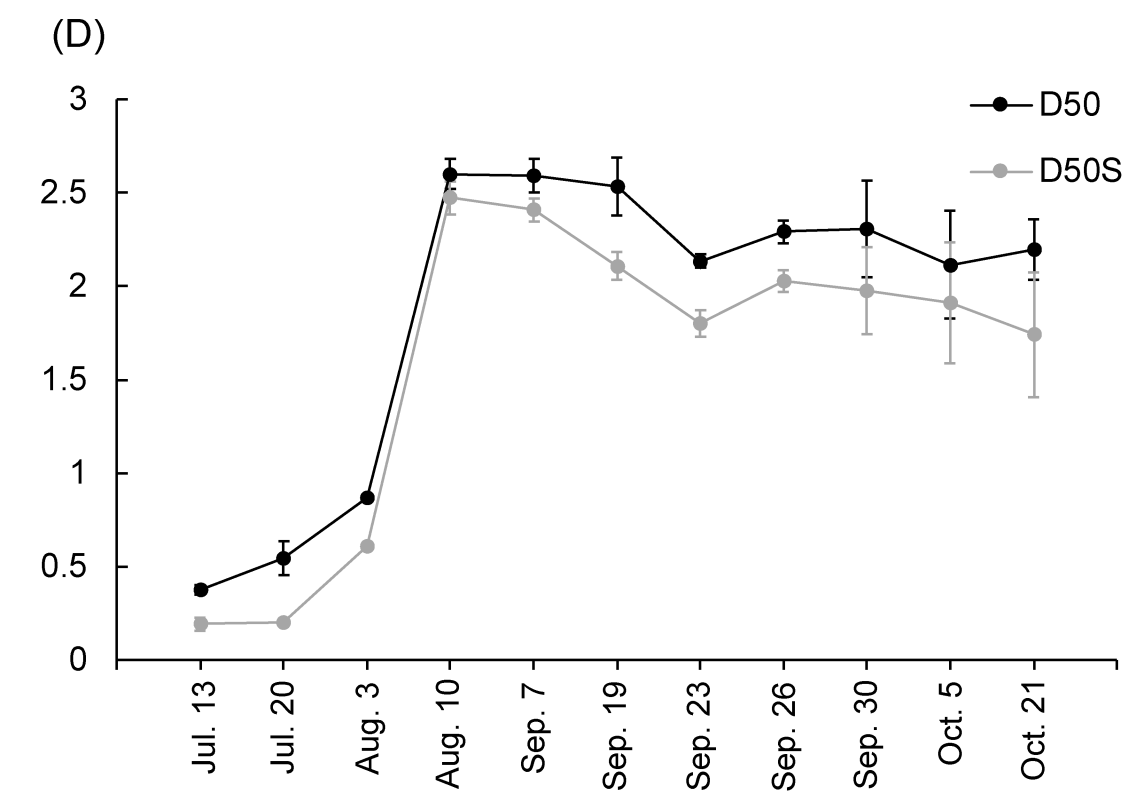

Sampling date

Supplement: Figure S5 — D0, D20, D30, D40, and D50 indicate the pond did not contain the artificial substratum, including 0, 20, 30, 40, and 50 individuals, respectively. D0S, D20S, D30S, D40S, and D50S indicate the pond contained the artificial substratum, including 0, 20, 30, 40, and 50 individuals, respectively. [file peerj-07-7906-s006.pdf]
